# New Insights on Ferroptosis and Gynecological Malignancies

**DOI:** 10.3389/fmolb.2022.921298

**Published:** 2022-06-14

**Authors:** Ruiqi Fan, Yujun Sun, Mengxue Wang, Qian Wang, Aifang Jiang, Tingting Yang

**Affiliations:** Center of Reproductive Medicine, Affiliated Hospital of Weifang Medical University, Weifang, China

**Keywords:** ferroptosis, gynecological malignancies, iron metabolism, reactive oxygen species, radiochemotherapy resistance, nanotechnology

## Abstract

Ferroptosis is a new type of cell death different from apoptosis and necrosis, which can regulate the accumulation of lipid peroxidation through different pathways, ultimately leading to cell death. An increasing number of studies have revealed that the relationship between ferroptosis and cancer is extremely complex, which holds promise as a new treatment. In gynecological malignancies, ferroptosis has been found to have excellent antitumor activity, which can regulate the proliferation, metastasis and radiochemotherapy resistance. With the continuous progress of research, nanodrugs, gene therapy and other new therapeutic techniques for inducing ferroptosis have been proposed. However, the study of ferroptosis in gynecological malignancies is still in its infancy, and further research is needed to design safe and effective cancer therapies based on ferroptosis. This article reviews the mechanism of ferroptosis and the latest research progress and prospects in gynecological malignancies.

## Introduction

Ovarian, cervical and endometrial cancer are the most common malignancies and the major causes of cancer-related mortality in women (Jin et al., 2021). At present, the treatments of gynecological malignant tumors are mainly surgical treatment combined with radiotherapy and chemotherapy, but the results are still not ideal because of its recurrence and drug resistance. Therefore, it is important to investigate the underlying molecular mechanisms and potential therapeutic targets associated with such tumors.

Ferroptosis is an iron-dependent cell death proposed by Dixon et al., in 2012 (Wang H. et al., 2020), characterized by the accumulation of reactive oxygen species (ROS) and lipid peroxidation (Zuo et al., 2020). This particular cell death pattern can be suppressed by lipophilic antioxidants and iron chelators (Chen et al., 2020). Morphologically, ferroptotic cells mainly manifest as rupture of the cell membrane and mitochondrial membrane, increased mitochondrial membrane density, reduced mitochondrial size, and decreased or disappeared mitochondrial ridge. While morphological changes in the nucleus are not obvious (Li et al., 2020). Ferroptosis has been found to play an important role in the pathogenesis and treatment of many diseases including nervous system diseases, ischemia reperfusion injury, various inflammatory disorders and cancers (Qiu et al., 2020). In recent years, many studies have shown that ferroptosis can not only inhibit the proliferation of ovarian cancer cells and their diffusion in the abdominal cavity (Basuli et al., 2017) but also reverse the chemotherapy resistance of ovarian cancer (Zhou et al., 2019). In addition, ferroptosis also plays an important role in the development and treatment of cervical cancer (Zuo et al., 2020) and endometrial cancer (Wang H. et al., 2021). Therefore, in-depth study of the ferroptosis will provide new opportunities for the treatment of gynecological malignant tumors.

## Mechanism of Ferroptosis

**FIGURE 1:**
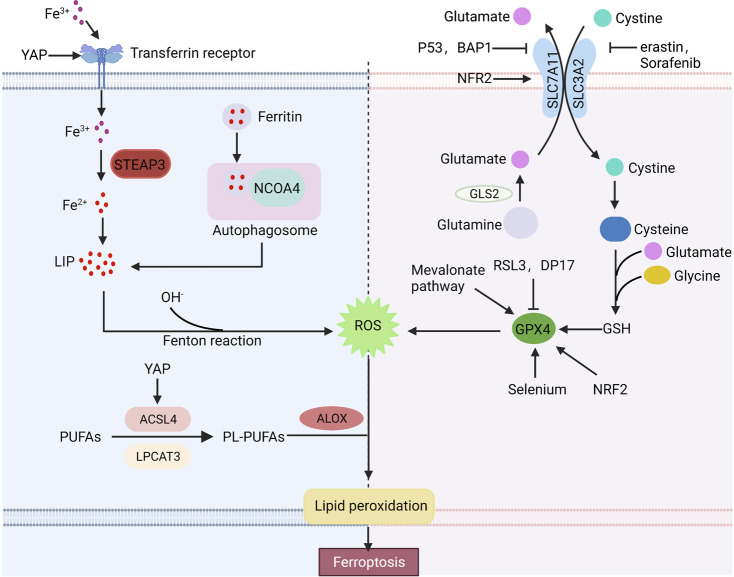
Basic ferroptosis pathways and regulators. Iron metabolism, the xCT-GPX4 pathway and lipid metabolism pathway are the primary metabolisms of ferroptosis. Furthermore, many factors can regulate ferroptosis. Arrows indicate positive effects, and perpendicular bars indicate negative effects.

Ferroptosis is a novel cell death mode, which requires excessive free iron and the accumulation of reactive oxygen species. Ferroptosis-inducing factors can reduce intracellular glutathione (GSH) levels and the activity of glutathione peroxidase 4 (GPX4) *via* different pathways, leading to the accumulation of ROS and ferroptosis (Figure 1).

### Iron Metabolism

The equilibrium state of iron is strictly controlled by iron metabolism in human body. Extracellular Fe^2+^ is oxidized to Fe^3+^ by ceruloplasmin, which binds to transferrin (TRF) and is transported into cells *via* transferrin receptor 1 (TFR1) (Frazer and Anderson, 2014). Then, Fe^3+^ is reduced to Fe^2+^ by six transmembrane epithelial antigen of prostate 3 (STEAP3) and stored in the unstable iron pool (LIP) or ferritin. The maladjustment of iron homeostasis may lead to ferroptosis (Bogdan et al., 2016). Excessive Fe^2+^ in cells can produce a large number of hydroxyl radical *via* the Fenton reaction. Hydroxyl radical has a strong oxidation ability to promote the accumulation of lipid peroxides, which leads to ferroptosis (Sun et al., 2021). The sensitivity of cells to ferroptosis can be influenced by regulating Fe absorption, storage and transport in human body. High TFR1 expression or increased ferritin autophagy affected by multiple factors and decreased ferritin expression can lead to excessive intracellular iron accumulation, thus improving cell sensitivity to ferroptosis. While low expression of TFR1 or overexpression of ferritin can reduce cell sensitivity to ferroptosis. It is reported that deletion of the transferrin receptor one gene (TFRC) reduces intracellular iron accumulation, while heme oxygenase promotes ferroptosis in cells by increasing intracellular iron accumulation (Kwon et al., 2015). Overexpression of heat shock protein family B (HSPB1) can downregulate TFR1 expression and reduce the intracellular iron concentration (Sun et al., 2015). In addition, researches have shown that inhibition of iron response element binding protein 2 (IREB2) increases the expression of ferritin and decreases the intracellular free iron concentration, thus inhibiting ferroptosis (Kim et al., 2021). Thus, regulation of iron metabolism, expression of iron transporters and intracellular iron concentration are additional potential points of ferroptosis.

### xCT-Glutathione Peroxidase 4 Pathway

The xCT system is an important component of the cellular antioxidant system, which is a cystine-glutamate antiporter composed of two subunits, SLC7A11 and SLC3A2. It pumps glutamate out of cells and cystine into cells at a 1:1 ratio, and cystine is an important raw material for the synthesis of intracellular glutathione (Bridges et al., 2012; Conrad and Sato, 2012; Koppula et al., 2018). Glutathione is widely present in cells and organelles. It can interact with glutathione peroxidase to eliminate lipid reactive oxygen species, thus maintaining cellular redox balance. Erastin, a small molecule of ferroptosis inducers, affects the synthesis of intracellular glutathione by inhibiting the xCT system, and decreases glutathione peroxidase activity, resulting in ROS accumulation and ultimately ferroptosis (Dixon et al., 2012). SLC7A11 is also an important regulatory site of ferroptosis. SLC7A11 overexpression enhances the antioxidant capacity of cells, thereby inhibiting erastin-induced ferroptosis (Huang et al., 2005). RSL3, a ferroptosis inducer, directly acts on GPX4 rather than the xCT system. GPX4, a member of the glutathione peroxidase family (GPXs), is a key regulator of ferroptosis. GPX4 catalyzes the reduction of lipid peroxides in complex cytomembrane environments. Researches have reported that downregulated GPX4 promotes tumor sensitive to ferroptosis, while upregulated GPX4 decreases ferroptosis sensitivity (Yang et al., 2014). In addition to inducers such as RSL3, DP17, the mevalonate pathway and selenium also act on the xCT system (Feng and Stockwell, 2018).

### Lipid Metabolism Pathway

Iron-dependent lipid peroxide accumulation is involved in all pathways of ferroptosis. Polyunsaturated fatty acids (PUFAs) in cells need to be embedded into membrane phospholipids under the catalysis of acyl-CoA synthase long chain family member 4 (ACSL4) and lysophosphatidyltransferase 3 (LPCAT3) (Lin et al., 2020). Researches indicate that knockdown of ACSL4 or LPCAT3 reduces PUFA phospholipid production to inhibit lipid peroxidation (LPO) deposition and erastin-induced ferroptosis (Nie et al., 2022), while ACSL4 overexpression promotes ferroptosis (Qiu et al., 2020).

### Important Regulators of Ferroptosis

Early studies have shown that p53 plays an important role in tumor suppression by inducing cell cycle arrest, senescence and apoptosis (Kaiser and Attardi, 2018). Recently, p53 has been found to be involved in the regulation of ferroptosis (Liu and Gu, 2022). p53 can downregulate SLC7A11 expression and affect intracellular glutathione synthesis, resulting in the accumulation of lipid peroxidation and ferroptosis (Jiang et al., 2015). In addition to affecting glutathione synthesis, SLC7A11 can bind to arachidonate 12-lipoxygenase (ALOX12) and reduce its enzyme activity (Chu et al., 2019). p53-mediated reduction of SLC7A11 can promote the release of ALOX12, which can play the role of oxide membrane PUFAs, eventually lead to ferroptosis (Liu and Gu, 2021). Moreover, p53 can promote the decomposition of glutamine by enhancing the activity of glutaminase 2 (GLS2). A high concentration of glutamate inhibits System xCT and induces ferroptosis (Hu et al., 2010; Suzuki et al., 2010). However, in some cases, p53 can inhibit ferroptosis by inducing p21 to converse GSH against ROS-mediated damage (Maddocks et al., 2013).

Nuclear factor E2-related factor 2 (NRF2) is considered to be an important inhibitor of ferroptosis, which can regulate the level of intracellular iron, limit the production of reactive oxygen species and upregulate the expression of SLC7A11 and GPX4 (Fan et al., 2017; Zimta et al., 2019; Takahashi et al., 2020). NRF2 activity is regulated by Keap1, which binds to NRF2 and inhibits NRF2 activity under normoxic conditions. However, NFR2 dissociates from Keap1 and transferred into the nucleus during stress conditions, where it activates antioxidant response elements (AREs) to maintain redox homeostasis (Fan et al., 2017).

The E-cadherin-NF2-Hippo-YAP/TAZ pathway plays an important role in regulating ferroptosis. High-density cells are insensitive to ferroptosis resulting in cysteine deficiency and GPX4 inhibition (Wu et al., 2019). E-cadherin (ECAD) is an important mediator of cell–cell contacts in epithelial cells and is highly expressed in dense cells (van Roy and Berx, 2008). In epithelial cells, ECAD inhibits YAP activity by inducing the intracellular NF2 and Hippo signaling pathways. YAP can promote ferroptosis by upregulation of several ferroptosis targets including ACSL4 and TFRC (Wu et al., 2019).

BRCA1-associated protein 1 (BAP1) is a tumor suppressor gene that promotes ferroptosis by downregulating SLC7A11, similar to p53. BAP1 downregulates SLC7A11 by H2Aub deubiquitination on SLC7A11, inhibiting cystine uptake and leading to lipid peroxidation and ferroptosis (Zhang et al., 2018).

## Ferroptosis in Gynecological Malignancies

Ferroptosis has been found to play an important role in the pathophysiological process of many malignant tumors. Studies confirm that inducing ferroptosis not only inhibits the growth of tumor cells but also enhances the sensitivity of tumor cells to chemoradiotherapy drugs in ovarian, cervical and endometrial cancers (Table 1).

**TABLE 1:** Ferroptosis and gynecological malignancies.

Tumor	Therapeutic target and drug	Mechanism of action	References
Ovarian cancer	Olaparib	Inhibit SLC7A11 expression by upregulating p53 to promote ferroptosis	Hong et al. (2021)
	SPIO	Synergize with p53 to promote ferroptosis	Zhang Y. et al. (2021)
	Erastin	Reduce the efflux transport activity of ABCB1 to reverse docetaxel resistance in ABCB1-overexpressing ovarian cancer cells	Zhou et al. (2019)
	Artesunate	Induce ROS accumulation to promote ferroptosis	(Shield et al., 2009; Greenshields et al., 2017)
	Ionizing radiation	Increase ROS accumulation and upregulate ACSL4 expression to promote ferroptosis	Zhang et al. (2022)
	Ferroptosis inducers	Inhibit SLC7A11 and GPX4 to enhance tumor cell sensitive to radiotherapy	Zhang et al. (2022)
	PD-L1 inhibitors	Activate CD8^+^ T cells to promote ferroptosis	Author Anonymous, (2019)
Cervical cancer	Oleanolic acid	Upregulate ACSL4 expression to promote ferroptosis	Xiaofei et al. (2021)
	Sorafenib	Increase iron concentration and ROS levels and decrease glutathione to promote ferroptosis	Wang C. et al. (2021)
	Artesunate-conjugated phosphorescence rhenium (I) complexes	Deplete glutathione, inactivate GPX4 and accunulate lipid peroxidatio to promote both apoptosis and ferroptosis	Ye et al. (2021)
	Ferroptosis inducers (sulfamazine)	Inhibit SLC7A11 and GPX4 to enhance tumor cell sensitive to radiotherapy	Lei et al. (2021)
Endometrial Cancer	Quinone	Regulate heme oxygenase, transferrin, SLC7A11 to promote ferroptosis	Zhang Y.-Y. et al. (2021)
	Inhibit PTPN18	Downregulate the activity of GPX4/xCT to promote ferroptosis	Wang H. et al. (2021)

### Ferroptosis in Ovarian Cancer

Ovarian cancer is the deadest gynecological malignant tumor. And the effective treatment is resection of all macroscopic tumors combined with chemotherapy. Although patients initially respond well to this treatment, 50% of patients relapse and develop drug resistance within six months (Lengyel, 2010). Multidrug resistance is considered to be a main cause of chemotherapy failure and a low 5-year survival rate in ovarian cancer, so eliminating the drug resistance of tumor cells is very important. It is revealed that iron efflux pump expression is decreased and transferrin receptor 1 (TFRI) is overexpressed in both high-grade serous ovarian cancer tissues and ovarian cancer tumor initiating cells (TICs), leading to intracellular free iron concentration increased (Basuli et al., 2017). Meanwhile, decreasing intracellular iron concentrations inhibits ovarian cancer cell proliferation and intraperitoneal dissemination. These suggest that ovarian cancer cells are highly iron-dependent in their growth, invasion and metastasis. This “iron addiction” increases ovarian cancer cell sensitivity to ferroptosis inducers such as erastin. So inducing ferroptosis in ovarian cancer could be a breakthrough for inhibiting tumor cell growth and metastasis.

p53 can directly act on the xCT system and inhibit cysteine-glutamate transporters to reduce intracellular glutathione production, thus leading to the accumulation of lipid reactive oxygen and ultimately increasing cell sensitivity to ferroptosis (Wang Y. et al., 2020). Although the mechanism of p53 regulation of ferroptosis in ovarian cancer cells is not fully understood, this target provides a new research direction for ovarian cancer treatment. PARP inhibitor olaparib in BRCA-mutant ovarian cancer targets this point. Olaparib inhibits SLC7A11 expression by upregulating p53 in ovarian cancer cells, and then affects glutathione synthesis, resulting in the accumulation of lipid peroxidation and ferroptosis (Hong et al., 2021). Ferroptosis inducers can enhance the sensitivity of BRCA-mutant ovarian cancer cells to PARP inhibitors *in vivo* and *in vitro* to inhibit the proliferation of tumor cell (Hong et al., 2021). This may provide a new strategy for the treatment of PARP inhibitors in BRCA-mutant ovarian cancer. In addition, p53-mediated ferroptosis contributes to the effects of metal-based drugs. For example, overexpression of p53 significantly promotes ferroptosis induced by superparamagnetic iron oxides (SPIO) in ovarian cancer cells, then inhibiting the growth of ovarian cancer (Zhang Y. et al., 2021). p53 mutation is associated with the clinical stage and progression of ovarian cancer. Previous studies confirmed that p53 mutation occurs in 96% of high-grade serous ovarian cancers and mutations in p53 and Kras promote ovarian cancer by transforming primary tubal epithelial cells into cancer cells in mice (Tarangelo et al., 2018). p53 mutation may promote the proliferation of ovarian cancer cells by reducing tumor sensitivity to ferroptosis.

The ferroptosis inducers erastin and sorafenib have been shown to inhibit tumor cell proliferation, metastasis and invasion in malignant tumors such as lung cancer and fibrosarcoma. The combination of ferroptosis inducers and chemotherapy drugs such as docetaxel and platinum drugs can improve the prognosis of patients by reducing chemotherapy resistance in ovarian cancer cells. ATP-binding cassette transporters subfamily B member 1 (ABCB1) is a multidrug resistance protein, overexpression of which is one of the main factors of cancer chemotherapy failure. Erastin reduces the efflux transport activity of ABCB1 to lead the accumulation of chemotherapeutical drugs in tumor cells. This reverses docetaxel resistance in ABCB1-overexpressing ovarian cancer cells (Zhou et al., 2019). Another study found that platinum-resistant ovarian cancer patients treated with sorafenib have significantly longer progression-free survival than those with placebo (Chekerov et al., 2018). However, erastin treatment over time can inhibit ferroptosis in tumor cells by upregulating cysteine biosynthase and decreasing lipid peroxidation (Seborova et al., 2019).

Artesunate, an antimalarial drug, inhibits the proliferation of tumor cells by inducing ferroptosis accompanied by the accumulation of ROS in cells. According to research findings, artesunate can induce ROS accumulation in ovarian cancer cells *in vivo* and *in vitro*, leading to ferroptosis and eventually inhibiting the proliferation of ovarian cancer (Greenshields et al., 2017). In addition, artesunate also restrains cancer peritoneal metastasis in a mouse model of ovarian cancer by inducing ferroptosis (Shield et al., 2009). Although the mechanism of these drugs in malignant tumors is still under investigation, it provides a novel direction for ovarian cancer treatment.

### Ferroptosis in Cervical Cancer

Cervical cancer, one of the most common gynecological malignancies, is mainly caused by human papillomavirus (HPV) infection (Cruz-Gregorio et al., 2021). In recent years, the morbidity and mortality rates of cervical cancer have declined because of early screening. Nevertheless, patients with local or distant metastasis have a poor prognosis due to limited treatment options. Studies on ferroptosis in cervical cancer are limited. ACSL4 is a ligase that synthesizes polyunsaturated fatty acid-containing phospholipids in the lipid metabolic pathway of ferroptosis, the deletion of which can inhibit ferroptosis by reducing lipid peroxidation (Kagan et al., 2017; Lei et al., 2020). In cervical cancer cells, oleanolic acid can induce ferroptosis by promoting ACSL4, while interfering with ACSL4 expression can reduce the inhibitory effect of oleanolic acid on tumor cell viability and proliferation (Xiaofei et al., 2021). Ferroptosis inducers also play an important role in cervical cancer proliferation and chemotherapy resistance. Sorafenib inhibits the growth of cervical cancer in mice by increasing iron concentration, ROS levels and decreasing glutathione (Wang C. et al., 2021). However, other research has reported that long-term using of the ferroptosis inducer erastin can promote HSPB1 expression in cervical cancer cells, which reduces lipid ROS and iron accumulation, and thus leads to erastin resistance. While inhibition of HSPB1 expression increases the anticancer activity of erastin in cervical cancer (Sun et al., 2015). In addition, researchers designed two artesunate-conjugated phosphorescence rhenium (I) complexes, which can induce both apoptosis and ferroptosis of cervical cancer cells by glutathione depletion, GPX4 inactivation and lipid peroxidation accumulation, improving the treatment efficiency (Ye et al., 2021). Further exploration of the mechanism of ferroptosis in cervical cancer may be significant for cervical cancer progression and treatment.

### Ferroptosis in Endometrial Cancer

The incidence and mortality rates of endometrial cancer have rapidly increased in recent years, and the prognosis of patients with metastasis or recurrence remains poor.

Currently, studies on ferroptosis in endometrial cancer are also increasing. Ferroptosis can be regulated by a variety of protein kinases. Protein tyrosine phosphatase nonreceptor type 18 (PTPN18) is associated with the occurrence and development of malignant tumors. PTPN18 expression is upregulated in endometrial cancer and inhibited ferroptosis by upregulating the activity of GPX4/xCT, thus promoting the growth of endometrial cancer cells (Wang H. et al., 2021). Quinones can not only lead to tumor cell death by regulating cell apoptosis and cycle arrest, but also lead to iron homeostasis imbalance by regulating iron metabolism in tumor cells. In endometrial cancer, quinone compounds inhibit endometrial cancer cell growth by inducing iron-dependent autophagy. It has been reported that quinone mediates the accumulation of free iron in endometrial cancer cells by regulating heme oxygenase, transferrin and SLC7A11, inducing ferroptosis (Zhang Y.-Y. et al., 2021). In a word, there are few studies on ferroptosis in endometrial cancer and more research is necessary. Ferroptosis plays an important role in endometrial growth and survival, and further study of its mechanism can provide new targets and strategies for the prevention and treatment of endometrial cancer.

## Prospects of Ferroptosis in Gynecological Malignancy Treatment

### Ferroptosis and Radiation Therapy

Radiation therapy is one of the main methods of treating gynecological malignant tumors, but radiation resistance is still the main factor of radiotherapy failure. In malignant tumors, ferroptosis inducers increase the sensitivity of tumor cells to ionizing radiation. Studies have shown that ionizing radiation can induce ovarian cancer cell ferroptosis by increasing ROS accumulation and upregulating ACSL4 expression. Besides, ionizing radiation also upregulates SLC7A11 and GPX4 to make tumor cells surviving resistant to radiotherapy (Zhang et al., 2022). Treating radioresistant ovarian cancer cells with ferroptosis inducers that inhibit SLC7A11 and GPX4 can enhance tumor cell sensitivity to radiotherapy. This phenomenon also occurs in cervical cancer that ferroptosis inducers enhance radiation efficacy by inhibiting SLC7A11 and GPX4 in a model of cervical cancer (Lei et al., 2021). Previous study found that sulfamazine alone has a poor effect on inducing ferroptosis in tumor cells, but it is less toxic and suitable for using *in vivo*. Combined with radiotherapy, sulfamazine can enhance the sensitivity of tumor cells to radiotherapy, which is expected to be a radiotherapy sensitizer for cancer treatment (Lei et al., 2020). These studies have great significance for the development of new drugs for sensitizing tumors to radiotherapy.

### Ferroptosis and Immunotherapy

Ferroptosis is involved in T cell-mediated antitumor immunity and affects tumor immunotherapy. Activated CD8^+^ T cells release interferon γ (IFNγ) to downregulate the expression of SLC3A2 and SLC7A11, and inhibit the uptake of cystine by tumor cells, thereby promoting lipid peroxidation and ferroptosis (Wang et al., 2019). Meanwhile, IFN-γ can also increase cell sensitivity to ferroptosis by upregulating the level of Fe^2+^ and downregulating the expression of GPX4 (Wei et al., 2022). Immunotherapy is used in combination with related therapies that induce ferroptosis such as targeted therapy, radiotherapy and chemotherapy, to achieve better therapeutic outcomes. A recent study showed that PD-L1 inhibitors can suppress tumor growth in ovarian cancer by activating CD8^+^ T cells, leading to the accumulation of lipid peroxidation and ferroptosis in the cells, and the combination with cystine/cysteinase showed stronger tumor inhibition (Author Anonymous, 2019).

### Ferroptosis, Nanomaterials, and Gene Technology

In recent years, some new methods, such as nanomaterials and gene technology, have been applied in malignant tumor treatments and chemotherapy resistance. Nanomaterials can use ultrasmall iron particles to release iron in malignant tumor cells, triggering the Fenton reaction to induce ferroptosis. Due to the complexity of malignant tumor treatment, the combination of multiple therapies can achieve better therapeutic effects. For example, chemotherapeutic drugs can be packed into ultrasmall particles of iron oxides to work together. A new ferroptosis inducer, sorafenib-mesoporous polydopamine-superparamagnetic iron oxide nanoparticles, which combine chemotherapy drugs, photothermal therapy and iron-based nanoparticles to inhibit the metastasis of malignant tumor cells (Guan et al., 2020). There are many similarly designed drugs, self-supplying lipid peroxidation nanoreactors that simultaneously release adriamycin, unsaturated lipids and iron, inducing ferroptosis (Zhu et al., 2022). These treatments can not only improve the effectiveness of malignant tumor but also reduce drug toxicity. In addition, gene technology can also be applied to gynecological malignant tumor, including gene knockout and gene transfection (Shen et al., 2018). p53, CBS and GPX4 are all functional gene loci mentioned above. It is of great significance to promote these studies and clinical applications in the treatment of gynecological malignant tumors.

## Conclusion

Ferroptosis is a new form of cell death, the role of which in malignant tumors has attracted extensive attention. The mechanisms of ferroptosis are very complex, and there are many other pathways besides the iron metabolism pathway, xCT-GPX4 pathway and lipid metabolic pathway mentioned above. Therefore, the mechanism of ferroptosis still needs to be further studied to provide more valuable treatments for diseases. At present, some key therapeutic targets in gynecological malignant tumors have been discovered, such as p53, CBS and GPX4. A number of small-molecule drugs have been designed for these targets to induce ferroptosis, but these drugs are not yet available in humans. Ferroptosis inducers can reverse chemotherapy resistance in gynecological malignant tumors, which is very beneficial for patients with advanced and chemotherapy-resistant gynecologic malignancies. Moreover, some less toxic ferroptosis inducers can be used as radiotherapy sensitizers, breaking the bottleneck of radiotherapy resistance. New technologies such as nanomaterials and gene technology are specific for gynecological malignant tumor treatment, but more experiments and researches are still needed. In conclusion, ferroptosis inducers are promising as emerging drugs in the treatment of gynecological malignant tumors, which are also the direction of our future research.

## Fundings

This research is supported by the National Natural Science Foundation of China (Nos. 81602301 and 81972489), Natural Science Foundation of Shandong Province (No. ZR2021MH235), Shandong province college science and technology plan project (No. J17KA254) and Clinical Research Center of Affiliated Hospital of Weifang Medical University (No. 2021wyfylcyj01).
